# The synergistic immunotherapeutic impact of engineered CAR-T cells with PD-1 blockade in lymphomas and solid tumors: a systematic review

**DOI:** 10.3389/fimmu.2024.1389971

**Published:** 2024-05-10

**Authors:** Bibhu Prasad Satapathy, Pooja Sheoran, Rohit Yadav, Dewan Chettri, Dhruba Sonowal, Chinmayee Priyadarsini Dash, Prachi Dhaka, Vivek Uttam, Ritu Yadav, Manju Jain, Aklank Jain

**Affiliations:** ^1^ Department of Zoology, Non-Coding RNA and Cancer Biology Laboratory, Central University of Punjab, Bathinda, Punjab, India; ^2^ Department of Biochemistry, School of Basic Sciences, Central University of Punjab, Bathinda, Punjab, India

**Keywords:** engineered CAR-T cells, PD-1, solid tumor, lymphoma, immunotherapy, combination immunotherapy

## Abstract

Currently, therapies such as chimeric antigen receptor-T Cell (CAR-T) and immune checkpoint inhibitors like programmed cell death protein-1 (PD-1) blockers are showing promising results for numerous cancer patients. However, significant advancements are required before CAR-T therapies become readily available as off-the-shelf treatments, particularly for solid tumors and lymphomas. In this review, we have systematically analyzed the combination therapy involving engineered CAR-T cells and anti PD-1 agents. This approach aims at overcoming the limitations of current treatments and offers potential advantages such as enhanced tumor inhibition, alleviated T-cell exhaustion, heightened T-cell activation, and minimized toxicity. The integration of CAR-T therapy, which targets tumor-associated antigens, with PD-1 blockade augments T-cell function and mitigates immune suppression within the tumor microenvironment. To assess the impact of combination therapy on various tumors and lymphomas, we categorized them based on six major tumor-associated antigens: mesothelin, disialoganglioside GD-2, CD-19, CD-22, CD-133, and CD-30, which are present in different tumor types. We evaluated the efficacy, complete and partial responses, and progression-free survival in both pre-clinical and clinical models. Additionally, we discussed potential implications, including the feasibility of combination immunotherapies, emphasizing the importance of ongoing research to optimize treatment strategies and improve outcomes for cancer patients. Overall, we believe combining CAR-T therapy with PD-1 blockade holds promise for the next generation of cancer immunotherapy.

## 1 Introduction

Despite recent breakthroughs in cancer diagnostics and therapeutics, cancer still remains the second biggest cause of death worldwide, with lung and colorectal cancer being two of the leading causes of death (1–3). There are plenty of substantial drawbacks to traditional treatment options, including surgery, radiation therapy, and chemotherapy, and many patients with metastatic or recurring ailments continue to witness poor outcomes (4). However, immunotherapies, particularly in the treatment of hematological malignancies, have demonstrated clinical success on a global scale (5). As a novel approach to cancer treatment, immunotherapy has heralded a new era of cancer therapy strategies (6). In contrast to traditional techniques, immunotherapy concentrates on using the body’s immune system to fight cancer. Immunotherapy helps to withstand and eliminate tumor cells by triggering the body’s natural defenses (7, 8). One such novel immunotherapeutic approach is adoptive cellular therapy (ACT) which majorly comprises T cell receptor (TCR) T cells, tumor-infiltrating lymphocytes (TILs), and chimeric antigen receptor T cells (CAR-T cells) (9, 10). Recently in 2023, FDA approved Lifileucel a type of TIL as a therapeutic option against unresectable or metastatic melanoma but its long-term efficacy needs to be studied (11). However, among all the available adoptive cellular therapies CAR-T cells have been widely studied over last decade and have shown promising results in various hematological and non-hematological malignancies (12–14).

Chimeric antigen receptor (CAR) T Cell therapy utilizes T-cells that have been genetically modified to possess unique tumor-killing abilities using CAR (15). This intriguing treatment strategy involves genetically modifying T-cells to produce distinct CARs, which enable the T-cell to identify and target a specifically selected and explored tumor antigen (16). In this regard, CD19, a particular antigen found in B cells, has been considered and applied in CAR-T cell therapy to treat lymphoma and leukemia (17). Some of the U.S. FDA approved CAR-T cell therapies are Yescarta (NCT02348216, NCT03105336), Kymriah (NCT02435849), Tecartus (NCT04880434), Breyanzi (NCT02631044), Abecma (NCT03361748), Carvykti (NCT03548207) under the generic name axicabtagene ciloleucel, tisagenlecleucel, brexucabtagene autoleucel, liscobtagene maraleucel, idecabtagene vicleucel and clitacabtagene autoleucel respectively. Of all these approved CAR-T cell therapies, axicabtagene ciloleucel, and tisagenlecleucel are approved for diffuse large B-cell lymphoma (DLBCL).

Although CAR-T cell therapy effectively treats recurrent or resistant hematological malignancies, treating solid tumors remains difficult. The complex tumor microenvironment (TME), aberrant vasculature, trafficking, tumor infiltration, and lack of reliable Tumor-Associated Antigens (TAAs) are some of the major problems with using CAR-T cells in solid tumors (18). To get around these challenges, various approaches have been utilized, such as giving CAR-T cells the ability to secrete cytokines or chemokines or to knock off PD-1 expression and utilizing CAR-T cells in combination with other therapies (19, 20). One of the potential problems with CAR-T cells is their exhaustion in the case of solid tumors, which refers to loss of effector function and persistent inhibitory receptor expression due to chronic antigen exposure and the immunosuppressive tumor microenvironment and immune checkpoint programmed death -1 (PD-1) is closely linked with CAR-T cell exhaustion (21–24).

PD-1 is a signaling receptor present on the surface of T-cells that regulates the activation of T-cells (25). Tumor cells can evade the immune system by preventing T-cell activation through upregulation of PD-L1/PD-L2 molecules, the ligand of PD-1. Therefore, some effective therapy is required to overcome this problem of CAR-T cell exhaustion in the case of solid tumors. Anti PD-1 therapy, along with CAR-T cell therapy, can help reverse exhaustion and increase the efficacy of CAR-T cells in case of solid tumors (26).

This review thoroughly explores the details of CAR-T cell structure, how it has evolved over different generations, and the involvement of the immune checkpoint PD-1, especially its signaling within solid tumors and lymphomas. The individual efficacy and impacts of CAR-T cell therapy and anti PD-1 drugs has been discussed. We present how the combination of these treatments performs, particularly when addressing solid tumors and lymphomas, adding depth to our understanding of their combined impact. To see the impact of combination therapy on different tumors and lymphomas, we categorized them based on six major tumor-associated antigens: mesothelin, disialoganglioside GD-2, CD-19, CD-22, CD-133, and CD-30, which are present in various tumors (27–32). In our opinion co-administration of both CAR-T cell and PD-1 inhibitor therapies simultaneously may lead to better outcomes, including enhanced progression-free survival, and improved overall survival rates.

## 2 Search strategy

**Figure 1:**
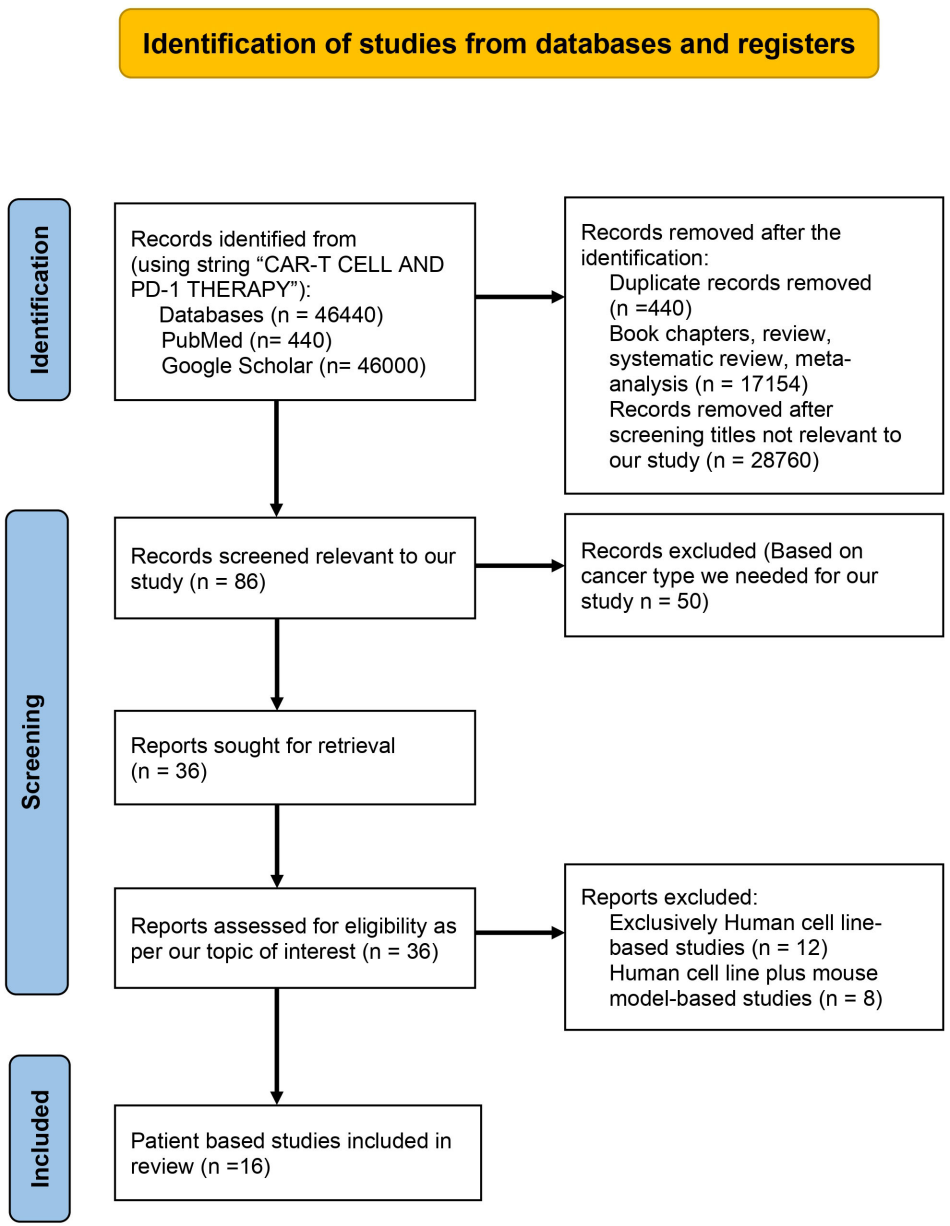
PRISMA flow chart describing the process of literature search and study selection related CAR-T cell therapy and PD-1 therapy in cancer.

The preferred reporting items for systematic reviews and meta-analysis (PRISMA) were used for the systematic analysis of literature we obtained after searching databases. For searching out the literature for our review, we considered authenticated databases such as Google Scholar and PubMed. No period constraint was applied during the search. We used the string “CAR-T CELL THERAPY” AND “PD-1 THERAPY IN CANCER”. After our primary research as of date 10/01/2024, we got 46440 papers, but after identification, we excluded duplicate records (n=440), reviews and book chapters (n=17154), and titles that did not consider combination therapy (n=28760). The records were reduced to 86 after identification and subjected to scientific screening. During the scientific screening, we removed 50 out of 86 records we obtained after identification based on the type of cancer we included in our review, and the records were reduced to 36. The human cell line-based studies (n=12) and human cell line plus mouse model-based studies (n=6) were also excluded after detailed screening. After the screening, the papers we had at the end were 16. As shown in **Figure 1**, these 16 papers were specifically chosen for inclusion in our review due to their focus on patient-based studies that were given a combination of CAR-T cell and PD-1 therapy. This detailed and systematic approach ensures that the literature synthesized in our review is not only substantial but also directly relevant to the clinical context of CAR-T cell therapy and PD-1 therapy in the context of cancer treatment.

## 3 CAR-T cell

CAR-T cells are genetically engineered T-cells that code and express customized receptors known as chimeric antigen receptors (CAR). These CARs are designed to efficiently recognize target tumor-associated antigens present in tumor cells (33, 34). Physiological T-cell typically require two signals for their activation; the first signal is provided by the interaction of MHC-bound antigen with T-cell receptor (TCR) and the second one is provided by the binding of costimulatory molecules such as CD28 (present on helper T-cells) and CD80 or CD86 (present on antigen-presenting cells) (35). In contrast to conventional T-cells, CAR-T cells possess the distinctive capability to recognize antigens independently of Major Histocompatibility Complex (MHC) molecules (35) which leads to potent T-cell activation and produces a strong, immediate, and long-term antitumor response (36). Moreover, CAR-T cells can persist in the body for several years, which contributes to its efficacy in eliminating cancer cells upon relapse. Melenhorst, et al. (2022) investigated the long-term persistence of CD-19-directed CAR-T cells in two chronic lymphocytic leukemia patients who achieved remission over ten years ago. They found that CAR T cells were still detectable after a decade with both patients while maintaining complete remission (37). This extraordinary efficacy has been particularly evident in treating leukemia and lymphoma. However, despite their success in hematological malignancies, CAR-T cells have faced many challenges in achieving satisfactory results in treating solid tumors (38). Thus, ongoing research endeavors are directed towards enhancing the therapeutic efficacy of CAR-T cells for solid tumors.

### 3.1 Structure of CAR-T

Chimeric antigen receptor (CAR) comprises four domains: an extracellular domain, a hinge domain, a trans-membrane, and an intracellular signaling domain (39, 40). The structure and function of each of these domains are described below:

#### 3.1.1 Extracellular domain

The extracellular domain of a CAR is constructed by combining the variable regions of heavy (VH) and light chain (VL) of antibodies (Ab) through a flexible linker, forming a single-chain variable fragment (scFv) (40, 41). Since CAR is composed of the antibody’s variable portion, it retains the antigen-binding properties and binds specifically to a target antigen. Notably, the scFv can recognize and bind to an antigen in a major histocompatibility complex (MHC)-independent manner, leading to the activation of CAR-T cells and the induction of potent antitumor activity (41). Extracellular domain determines the CAR’s affinity for the target tumor-associated antigen; studies have shown that a higher affinity of the scFv for the target antigen can reduce on-tumor off-target effects. However, excessively higher affinity may compromise the tumor penetration ability and trigger unwanted toxicity (42). Thus, while designing a CAR, careful consideration of the affinity of the extracellular domain toward the target tumor antigen is essential to optimize its therapeutic efficacy while minimizing potential adverse effects.

#### 3.1.2 Hinge domain

The hinge domain is part of an extracellular region that links the transmembrane domain and the extracellular antigen-binding domain in a CAR. This region is usually derived from CD8, CD28, IgG1, or IgG4 (43, 44) and provides flexibility and helps to overcome steric hindrance, which is required to enhance the binding affinity of scFv with the target antigen (40, 43–45).

The extracellular region derived from IgG1, of the CAR bears a resemblance to the Fc region of antibodies. Consequently, immune cells with Fc gamma receptors (FcγR) can bind to the CAR’s extracellular region through Fc-FcγR interaction (46). This interaction has been observed to accelerate the aging process of CAR-T cells, resulting in a negative impact on CAR-T cell persistence and function (46). To mitigate this Fc-FcγR interaction, increasing the flexibility of the hinge domain becomes crucial. Therefore, when designing a CAR, careful consideration of the hinge region’s flexibility is essential to optimize CAR-T cell performance and longevity.

#### 3.1.3 Transmembrane domain

The transmembrane domain facilitates the connection between the intracellular and the extracellular domain through the hinge domain and transmembrane region is usually derived from proteins such as CD3ζ, CD28, CD4 and CD8α (41). CAR-T cells containing transmembrane domains derived from each of these proteins are known to release different levels and types of cytokines. Leah et al. (2017) evaluated the CD8α and CD28 transmembrane and hinge domain containing CAR-T cells, and they found that CAR-T cells containing CD8α released fewer cytokines (such as IFN-γ and TNF) as compared to CD28 containing CAR-T cells. They also found that the CAR containing transmembrane domain derived from CD8α showed lower activation induced cell death (AICD) as compared to a CAR containing the same regions derived from CD28 (47). Therefore, the choice of transmembrane domain plays an important role in determining the persistence of CAR-T cells.

#### 3.1.4 Intracellular signaling domain

The intracellular signaling domain is composed of the activation and costimulatory domains. The intracellular domain tries to replicate the typical sequence of events that triggers T-cell activation. The activation domain of CAR is usually derived from CD3ζ, which can activate CAR-T cells to some extent, but full activation requires extra costimulatory domains (48). These costimulatory domains are usually derived from CD28 or 4-1BB(CD137) (41, 43). CAR-T cells having either of these domains will have different distinct functions, CAR-T cells containing CD28 will differentiate into effector memory cells, while CAR-T cells containing 4-1BB (CD137) will differentiate into central memory T-cells (49). In general, upon binding with the target antigen, CAR-T cells containing activation and costimulatory domain produce IL-2, which helps in its proliferation and persistence (43, 50).

### 3.2 Generations of CAR-T cells

**Figure 2:**
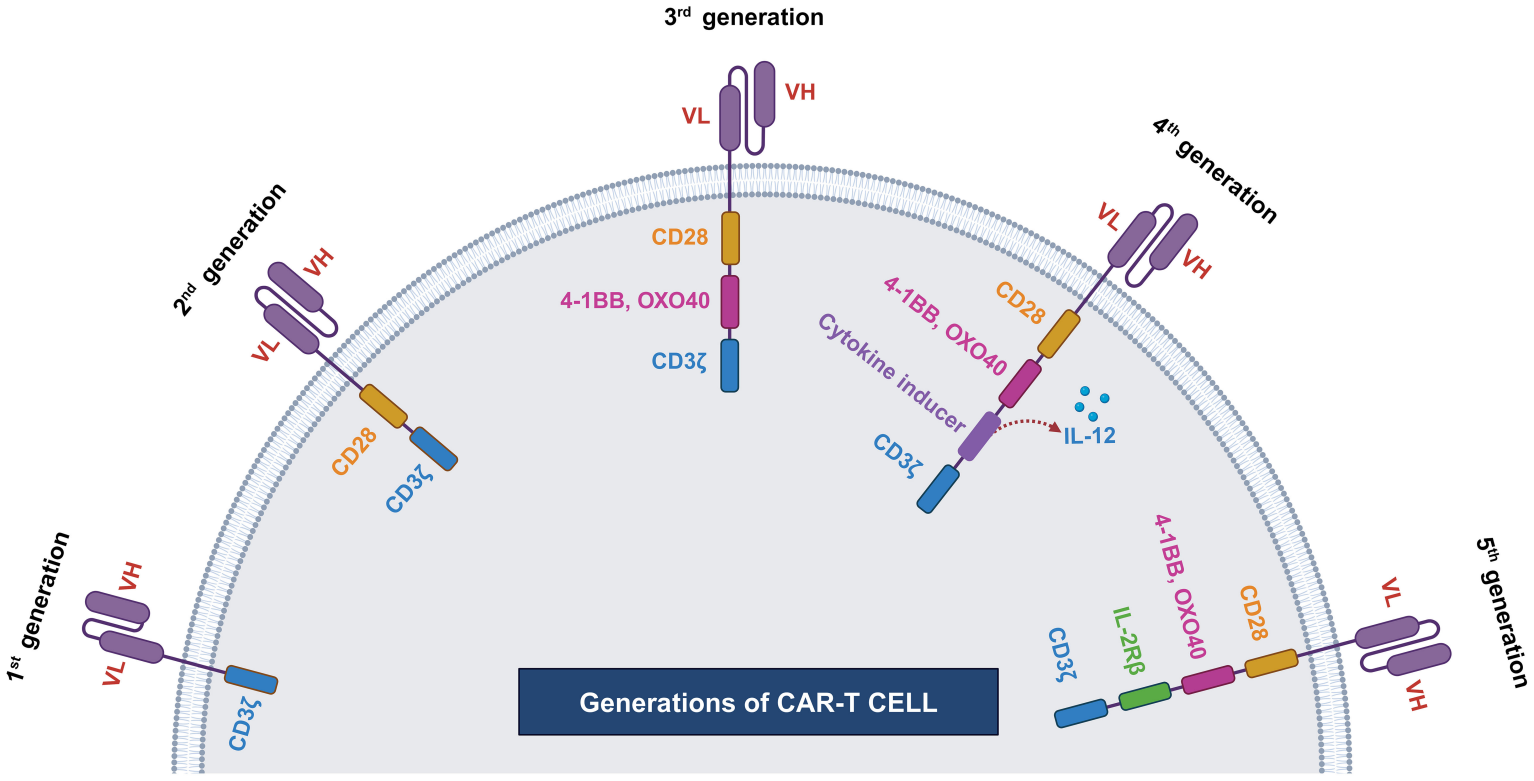
Generations of CAR-T cells. First generation of CAR-T cell has single cytoplasmic CD3ζ domain, second generation CAR-T includes CD3ζ along with costimulatory domain (CD28). Third generation CAR-T cell has multiple costimulatory domains, such CD3ζ, OXO40 or 4-1BB, and CD28. Fourth generation CAR-T contains additionally cytokine inducer domain and fifth generation CAR-T cell contains IL2R domain.

The initial concept of the Chimeric Antigenic Receptor (CAR) was first articulated in 1987 by Yoshihisa Kuwana and his colleagues at Fujita Health University and Kyowa Hakko Kogyo (51). Concurrently, Gideon Gross and Zelig Eshhar independently described the same concept in 1989 at the Weizmann Institute (52). The pioneering clinical application of CAR-T cell therapy was carried out at the University of Pennsylvania and the Children’s Hospital of Philadelphia under the leadership of Carl June. Their team successfully treated a five-year-old girl who was suffering from Acute Lymphoblastic Leukemia (53). Since then, various enhancements have been made to increase the efficacy of CAR-T cells. Mostly these enhancements are done by modifying the endodomains. Based on the structure and composition of the endodomain, CAR-T cells can be classified into five generations. We have shown the evolution of CAR-T cell generations in **Figure 2** (54).

#### 3.2.1 First generation

The first-generation CAR-T had a single intracellular signaling domain, such as CD3ζ or FcϵRIγ, but lacked costimulatory domains, such as CD27, CD28, CD134, and CD137 (55). These intracellular domains were responsible for transmitting signals from T-cell receptors, but CAR-T cells containing only intracellular domains had some limitations. The primary limitations were low cytotoxicity and proliferative responses. Thus, to overcome this limitation, exogenous IL-2 had to be supplemented to enhance their proliferative and cytotoxic capabilities. However, even after exogenous administration of IL-2, CAR-T showed limited proliferation and cytotoxicity, necessitating the development of second-generation CAR-T with additional costimulatory domains (55, 56).

#### 3.2.2 Second generation

The development of second-generation CAR-T cells aimed to overcome the limitations of first-generation, such as insufficient cytokine production such as IL-2 and a short lifespan. These challenges were effectively addressed by incorporating a dual signaling domain that promotes robust T-cell proliferation and differentiation into effector cells.

This achievement involved two types of signals. The first signal occurs when a foreign peptide binds to an MHC protein on the surface of an antigen-presenting cell, triggering recognition by a T-cell receptor and downstream signaling through CD3ζ. The second signal is initiated by the interaction between the CD28 co-receptor protein on the T-cell and the CD80/86 costimulatory protein on the antigen-presenting cell (57). Together, these signal 1 and signal 2 events stimulate T-cells to release IL2, which, in an autocrine manner, promotes their proliferation and differentiation (56–59).

Notably, a CAR with only CD3ζ is insufficient for full T-cell activation; the inclusion of a costimulatory domain is also required. Therefore, second-generation CARs incorporated CD28, 4-1BB, or OX-40 costimulatory domains. This design has been associated with improved T-cell proliferation, enhanced cytotoxicity, sustained response, and increased survivability of CAR-T cells (56, 60).

#### 3.2.3 Third generation

Third-generation CAR-T cells were developed by incorporating multiple costimulatory signaling domains along with the endodomain. Commonly utilized configurations included CD3ζ-CD28-OX40 or CD3ζ-CD28-41BB (61, 62). In a pilot clinical trial conducted by Brain et al. (2012) to assess the effectiveness of third-generation CAR-T cells containing CD28 and 4-1BB costimulatory domains, the study revealed improved persistence, proliferation, and safety (63). However, there were no significant enhancements in efficacy observed.

#### 3.2.4 Fourth generation

The development of fourth-generation CAR-T cells aimed to enhance the antitumor response of CAR-T by delivering transgenic products to the tumor site, thereby recruiting other immune cells (64). This advancement involved re-engineering third-generation CAR-T cells by incorporating an additional nuclear factor of the activated T-cell (NFAT) responsive cassette (65). This cassette allowed for the inducible expression of a transgenic cytokine, such as IL12. Commonly referred to as T-cell redirected for universal cytokine-mediated killing (TRUCK), the fourth-generation CAR-T exhibited improved activation and persistence (65).

#### 3.2.5 Fifth generations

To address challenges associated with fourth-generation CAR-T cells, the fifth-generation CAR-T cells were developed. Along with conventional CD3ζ domain fifth generation CAR-T has additional IL-2Rβ and STAT3 domain (46, 62, 66). These supplementary domains provide fifth-generation CAR-T cells with enhanced T-cell activation, proliferation, and improved properties for infiltrating solid tumors. Notably, unlike conventional monovalent CAR-T cells, fifth-generation CAR-T cells are designed to be multivalent, enabling them to recognize a broader range of tumor-associated antigens (62).

### 3.3 Clinical efficacy of CAR-T cell therapy

Various clinical studies have been done to determine the efficacy of CAR-T monotherapy, and it was found that CAR-T cell treatment against hematologic malignancies showed remarkable success. In contrast, in the case of solid tumors, the clinical efficacies were less than optimal (67). Some of the primary clinical trials are shown in **Table 1**. One of the reasons for low efficacy is the heterogeneous nature of the Tumor Microenvironment (TME) of solid tumors that presents unique challenges such as inadequate CAR-T cell infiltration, limited persistence, and suboptimal trafficking (68). It is well-documented that the PD-1/PD-L1 axis significantly limits the effector function of CAR-T cells (69). Therefore, blocking the PD-1/PD-L1 axis represents a promising approach to enhance the antitumor activity of CAR-T cells (70). The PD-1/PD-L1 interaction mechanism and how to target this axis to increase the effectiveness of CAR-T cell therapy have been covered in the subsequent sections.

**Table 1:** List of clinical trials on standalone CAR-T cell therapy conducted by various institutes or companies for solid tumor and lymphoma.

NCT Number	Company/Institute	Cancer type	CAR-T type	Trial stage	Started date	Status
NCT02107963	National Institutes of Health Clinical Center, US	refractory neuroblastoma	Anti GD2 CAR-T	Phase 1	02-02-2014	Completed
NCT04483778	Seattle Children’s Hospital, US	relapsed or refractory non-CNS solid tumors	B7H3 CAR-T cell	Phase 1	13-07-2020	Undergoing
NCT04377932	Texas Children’s Hospital, US	pediatric solid tumors	Glypican specific CAR-T	Phase 1	08-12-2021	Undergoing
NCT05274451	University of California, US	relapsed or refractory triple negative breast cancer	ROR1-targeted, CAR-T	Phase 1	29-03-2022	Undergoing
NCT03618381	Seattle Children’s Hospital, US	relapsed or refractory non-CNS solid tumors	EGFR specific CAR-T	Phase 1	18-06-2019	Undergoing
NCT02706392	University of Washington Cancer Consortium, US	Acute Lymphoblastic Leukemia, Triple negative Breast Cancer.	ROR1 specific CAR-T	Phase 1	16-03-2016	Completed
NCT01822652	Houston Methodist Hospital and Texas Children Hospital, US	relapsed or refractory neuroblastoma	GD2 specific CAR-T	Phase 1	08-2013	Undergoing
NCT05035407	National institute of Health Clinical Center, US	Gastric, Cervical, Lung, Breast and other KK-LC-1 positive Cancer	KK-LC-1 Specific CAR-T	Phase 1	08-03-2022	Undergoing
NCT02274584	University of Florida and Peking University Cancer Hospital, Beijing, China	Lymphoma	Anti-CD30 CAR T	Phase 1 and Phase 2	03-2014	NA
NCT02917083	Houston Methodist Hospital, Texas, United State and Texas Children’s Hospital	Lymphomas	Anti-CD30 CAR T	Phase 1	08-05-2017	Undergoing
NCT04260932	Hebei Yanda Ludaopei Hospital, China	refractory and relapsed B-Cell Lymphoma	CD19/CD20 Dual- CAR-T Cells	Phase 1	01-03-2020	Completed

## 4 Immune checkpoint PD-1 and its role in T cell regulation

Immune system checkpoints are molecules that regulate immunological responses, such as T-cell activation, its inhibition and preventing autoimmunity (71). In recent decades, several inhibitory immunoreceptors, such as programmed cell death protein 1 (PD-1; CD279), PD-L1 (CD274; B7-H1), cytotoxic T-lymphocyte associated protein 4 (CTLA-4; CD154), T cell immunoglobulin and mucin domain-containing protein 3 (TIM3), and lymphocyte-activation gene 3 (LAG3; CD223), T-cell immunoreceptor with Ig and ITIM domains (TIGIT), and B and T lymphocyte attenuator (BTLA; CD272), have been identified and are being investigated in relation to cancer (72, 73). Among all these, anti PD-1/PD-L1 therapy is one of the most studied immune checkpoint blockade therapies in cancer, and it has been approved to treat a wide range of cancer types, including blood, skin, lung, liver, bladder, and kidney cancers (74). CAR-T cell treatment is effective against hematologic malignancies but encounters limitations in treating solid tumors due to the expression of checkpoint PD-1/PD-L1 within the tumor microenvironment (TME), which directly limits T-cell responses (75, 76).

### 4.1 PD-1

Programmed cell death protein 1 (PD-1) or CD279 is a cell surface receptor protein known to act as a crucial immunosuppressive molecule. When stimulated, PD-1 is expressed by Natural killer T-cells, helper T-cells, Cytotoxic T-cells, B-cells, and activated monocytes (77). Immune checkpoint receptor PD-1 has two ligands, PD-L1 (B7- H1) and PD-L2 (B7-DC), which help in immune tolerance (78). PD-L1 (programmed cell death ligand 1) binds to PD-1 and checks the downstream signaling of T cell receptors. PD-1 also prevents T cells from activating and proliferating and makes it possible for tumor cells to evade the immune system (79, 80). Activated T-cells express PD-1 on their surface and are also responsible for the secretion of interferon γ, which stimulates tumor cells to express PD-L1 (81). The binding of PD-1 to its ligand restricts T-cell activation (82). PD-1 encourages immune tolerance when activated by PD-L1, which is mostly expressed in tumor microenvironments (83). Upregulating surface PD-L1 allows tumor cells to adjust PD-1-mediated inhibitory signaling (84).

### 4.2 Structure of PD-1

In humans, PD-1 is encoded by PDCD1 gene which is located on chromosome 2 (85). PD-1 receptor is a 55 kDa transmembrane glycoprotein of 288 amino acids, which consists of an extracellular immunoglobulin variable-type amino-terminal domain (147 amino acids), a stalk of around 20 amino acids that acts as a spacer between extracellular domain and the cell’s plasma membrane (86, 87). There is a transmembrane region which consists of 27 amino acids. The cytoplasmic signaling domain consists of 94 amino acids that contain an immunoreceptor tyrosine-based inhibitory motif (ITIM) and an immunoreceptor tyrosine-based switch motif (ITSM), necessary for the inhibitory function of TCR signaling (88, 89).

### 4.3 Anti PD-1 therapy in solid tumors

PD-1 expression is elevated in T-cell within the tumor microenvironment than those found in healthy tissues (90). Therapies that involved inhibition of immune regulatory checkpoints, i.e., PD-1, PD-ligands (PD-L1, PD-L2), and CTLA-4 have shown remarkable success in a broad range of hematological malignancies and solid tumors, leading to a substantial enhancement in overall survival rates. Immune checkpoint inhibitors have become primary therapies for various types of cancers, including metastatic melanoma and non-small cell lung cancer (91, 92). Approximately 30% of solid tumor types and hematologic malignancies exhibit overexpression of PD-L1 to block antitumor immune responses and enhance tumor growth, proliferation, and survival (87). Many human cancers have PD-L1 overexpression, including melanoma (40%-100%), NSCLC (35%-95%), and nasopharyngeal carcinoma (68%-100%). urothelial carcinoma (28%-100%) lymphomas (17%-94%), and others (87). Blackburn et al. (2009) demonstrated that colon cancer patients with mismatch repair (MMR) deficiency accumulate a higher tumor mutational burden, leading to increased neoantigen production and enhanced recognition by the immune system. These colon cancer patients respond better to anti PD-1 therapy. These findings suggest that immune checkpoint inhibitors may be useful for various cancers (87). Malignant melanoma, a challenging skin cancer to treat, responds favourably to immunotherapy treatments such as PD-1/PD-L1 inhibitors (93). A significant proportion of melanoma malignancies, roughly 38% of all cases, have positive tests for PD-L1 and TILs, making them prime candidates for blockade by PD-1/PD-L1 inhibitor (94). Breast cancer treatment using mAbs against PD-1/PD-L1 is showing promising results. PD-L1 expression rates are high in triple negative breast cancer (TNBC), estrogen-negative, and progesterone-negative tumors. PD-1/PD-L1 inhibitors have been found to exhibit a response rate of 19% in patients with triple-negative breast cancer (TNBC) (95–97).

### 4.4 Molecular mechanism of PD-1 blockade in T-cells

**Figure 3:**
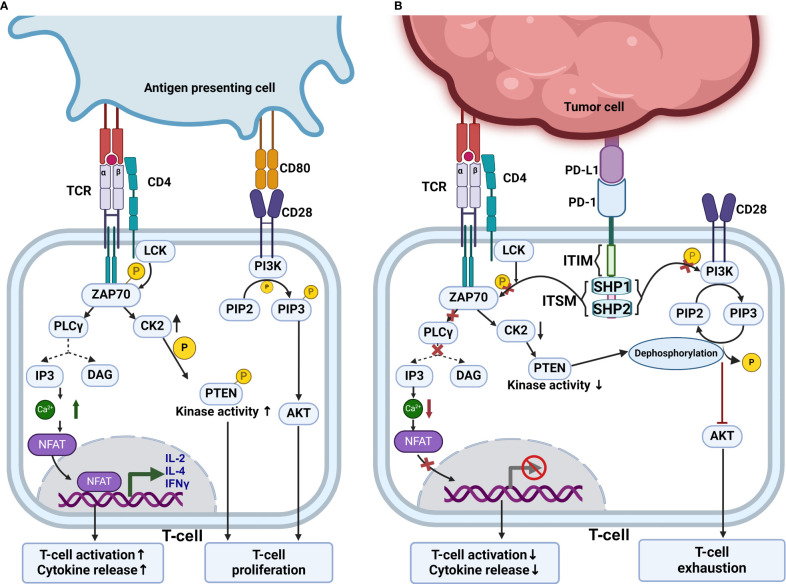
PD-1 blockade mechanism. **(A)** Mechanism of normal T cell activation which is achieved by interaction of T cell receptor with MHC bound antigens and CD80-CD28 costimulatory interaction. This normal T cell activation leads to upregulation of CK2 which phosphorylates PTEN inhibiting its phosphatase activity. This allows the PI3K-Akt pathway to activate T cells. **(B)** Inhibitory mechanisms of PD-1, which depend on the recruitment of SHP1 and SHP2 phosphatases. These phosphatases inhibit ZAP70 and PI3K activities. Terminating downstream intracellular signaling pathways. Also, PD-1 indirectly regulates T cell proliferation by targeting PTEN and inhibiting PI3K-Akt pathway. When CK2 is downregulated PTEN is no longer phosphorylated and shows phosphatase activity and dephosphorylate PIP3. This inhibits PI3K-Akt pathway and leads to T cell exhaustion.

T-cell activation and its effector function are regulated by multiple positive and negative signals alongside co-stimulatory and co-inhibitory signals. The positive signal is provided by the interaction of CD80 with CD28, and the negative signal is provided by the interaction of immune checkpoint inhibitors such as CTLA4 and PD-1 (98). During T-cell activation, downstream signaling is initiated by recruiting and activation of src-like tyrosine kinases, mostly LCK. LCK phosphorylates the intracellular domain of CD3 and TCR, which leads to the recruitment of ZAP-70 and PI3K to CD3 and CD28 (99). Once ZAP-70 is recruited and activated, it goes on to activate PLCγ. This causes IP3 mediated release of Ca2+ from the endoplasmic reticulum and this also causes translocations of NFAT and CREB into nucleus. These transcription factors cause transcription of genes involved in T-cell differentiation (100). Full activation and differentiation of T-cells require an additional co-stimulatory signal, which is provided by the CD80-CD28 association. This causes the production of PIP3, which is required for AKT and PKC-θ activation (101); this leads to strong activation and proliferation of T-cell as illustrated through **Figure 3A**. Moreover, T-cell activation, proliferation, and differentiation can be inhibited by PD-1/PD-L1 interaction (102).

The expression of PD-1/PD-L1 in cancer cells is influenced by various pathways such as PI3K/AKT, MAPK, JAK/STAT, Wnt, NF-κB, and Hedgehog (Hh). The phosphoinositide 3-kinase (PI3K)/Akt pathway, vital for cellular metabolism control, is a major target of PD-1-mediated inhibition in T-cells (87, 103).

Understanding the complex nature of the PD-1/PD-L1 pathway has led to revolutionary advancements in oncology and immunology, revolutionizing the treatment of various cancers and autoimmune conditions (104). This pathway’s association is crucial for regulating immune responses to prevent excessive activation, thereby averting autoimmunity and inflammation. However, certain diseases exploit this mechanism to evade immune detection. For instance, cancer cells increase PD-L1 expression, which binds to PD-1 on T-cell and dampens their ability to combat tumors (91).

The cytoplasmic tail of PD-1 contains two tyrosine residues, one within an ITIM motif near the cell membrane and another in ITSM (105). When PD-1 is activated by either of its natural ligands, PD-L1 or PD-L2, Src kinases phosphorylate PD-1 at two tyrosine-containing motifs, ITIM and ITSM (106), which recruit and activate phosphatase such SHP1 and SHP2, removing phosphate groups from nearby effector proteins CD3ζ, ZAP70, and PI3K kinases (107). This leads to the suppression of phosphoinositide 3-kinase (PI3K)–Akt pathway promoting T-cell exhaustion (108). Usually, in the absence of PD-1 activation, TCR leads to increased expression of CK2, which phosphorylates and inactivates PTEN. This inactivated PTEN can no longer dephosphorylate PIP3, which is produced by PI3K. These allow TCR signal transduction to proceed and activate T-cells (109). Meanwhile, if the PD-1 is engaged with PD-L1, CK2 kinase expression is down-regulated. When the CK2 level is downregulated, PTEN gets activated; thus, PIP3 gets dephosphorylated, blocking downstream signaling (110). Thus, by indirectly inhibiting T-cell activation, PD-1 influences T-cell proliferation as demonstrated in **Figure 3B**. The development of immunotherapies like immune checkpoint inhibitors targeting PD-1 or PD-L1 has significantly improved T-cell proliferation, tumor infiltration, survival, and efficacy against specific cancers (83).

## 5 CAR-T cell and anti PD-1 combination therapy

Previous research has shown that CAR-T and PD-1 blockade therapies have individually shown clinical success and the FDA has approved a few drugs, their efficacy against solid tumors is still limited and needs further study and improvement (111). They are also showing good results against certain lymphomas but there are risks of resistance, recurrence, cytokine release syndrome, and neurotoxicity (112, 113). Tumor escape mechanism, a hallmark of cancer, are often facilitated by the expression of checkpoint ligands on tumor surfaces (114). Activated T-cells naturally upregulate co-inhibitory receptors such as PD-1 and CTLA-4 upon activation (41). The solid tumor shows higher expression of co inhibitory ligands such as PD-L1 (114) that bind to T-cell inhibitory receptors (115). Under normal physiological conditions, the PD-1/PD-L1 pathway maintains peripheral immune tolerance and prevents excessive tissue inflammation and autoimmune diseases (116). However, within the tumor microenvironment, these receptors compromise the host’s anti-tumor immunity by inducing immune suppression by inhibiting tumor-infiltrating Lymphocytes (TIL) activation, cytotoxic T lymphocyte (CTL) production, cytokine secretion, and thus promote tumor progression and metastasis (116). These ligands also limit the efficacy and persistence of CAR-T cell therapy (71, 115, 117). To overcome this limitation, combination therapy of CAR-T cells along with anti PD-1 has the potential to be the next generation of cancer immunotherapy for the treatment of solid tumors.

### 5.1 Types of combination therapy: extrinsic and intrinsic

The combination therapy can be of two types: Cell extrinsic and cell intrinsic combination therapy.

#### 5.1.1 Cell extrinsic combination therapy

In the extrinsic method, the PD-1 blocking agents are administered separately before or after the CAR-T cell treatment. Currently, at least three such anti PD-1 monoclonal antibodies (mAb) are approved by FDA. Namely, Nivolumab (Opdivo), Pembrolizumab (Keytruda), and Cemiplimab (Libtayo), several anti PD-1 agents are under trial (118). PD-1 mAbs tend to promote the secretion of IL-2 and IFN-γ (119, 120). IL-2 acts as a T-cell growth factor; thus, it was amongst the first immunotherapeutic drugs to get FDA approval nearly three decades ago for cancer treatment (121). IFN-γ has shown to reduce the immune suppression that occurs at times and reduce tumor burden (122). Thus, combining these anti PD-1 agents with CAR-T cells can significantly increase the persistence and anti-tumor effect of CAR-T cells. In an *in vivo* study by John, L.B. et al. (2013) in an HER-2+ mice model, CAR-T cells combined with an anti PD-1 antibody were administered. PD-1 antibody significantly increased the level of IFN-γ, granzyme-B, and a proliferation marker of T-cell that is Ki-67; the CAR-T cell combined with anti PD-1 showed significantly higher anti-tumor response compared to only CAR-T cells and isotype control antibody (123). However, the optimal doses and frequencies of administration of anti PD-1 may not be generalized as different targets for immunotherapy and different agents show different dose response characteristics (124). Thus, in the case of cell extrinsic combination therapy, the doses of PD-1 blocking agents vary based on tumor type and grading, and there may be a need for repeated administration of anti PD-1 antibodies at a regular interval. For example, in a phase I study, Adusumilli et al. (2021) used Pembrolizumab as an extrinsic PD-1 blockade along with mesothelin-specific CAR-T cells in a group of 16 patients with malignant pleural mesothelioma (MPM). They observed a higher persistence of CAR-T cells in the combination group than in the group that received standalone CAR-T cell therapy. They have administered multiple Pembrolizumab doses, which varied from 1 to as high as 30 doses (125). Although extrinsic combination therapy shows promising results, there are some drawbacks associated with it. Kuah et al. (2023) suggested optimizing the doses of anti PD-1 drugs as current administration regimens may result in overtreatment with potentially important implications for cost, quality of life, and toxicity (124).

#### 5.1.2 Cell Intrinsic combination therapy

In the cell intrinsic method of combination therapy, the CAR-T cells are genetically engineered to have anti PD-1 activity. One of the methods to create cell intrinsic CAR-T cells is by knocking out genes that are responsible for PD-1 expression, such as *PDCD1* on CAR-T cells using various genome editing tools such as CRISPR-Cas9 or TALEN and then the recombinant DNA is transduced using viral vectors (126) or electroporation (127) into the autologous T-cell. PD-1/PDL-1 axis is responsible for the modulation of immune tolerance (128). Genetic disruption of PD-1 may pose a chance of potential autoimmune response and higher chances of immune related adverse events. Wang, et al. (2021) studied the efficacy of intrinsic CAR-T cells by knocking out *PDCD-1*. To reduce the chances of GVHD, they also knocked out the T-cell receptor alpha constant (TRAC) using CRISPR-Cas9 and produced MSLN-directed 28ζ CAR-T cells with PD-1 and TCR disruption (MPTK-CAR-T cells). From the study it was found that using CRISPR-edited CAR-T cells with PD-1 disruption was viable and secure to be used.

**Figure 4:**
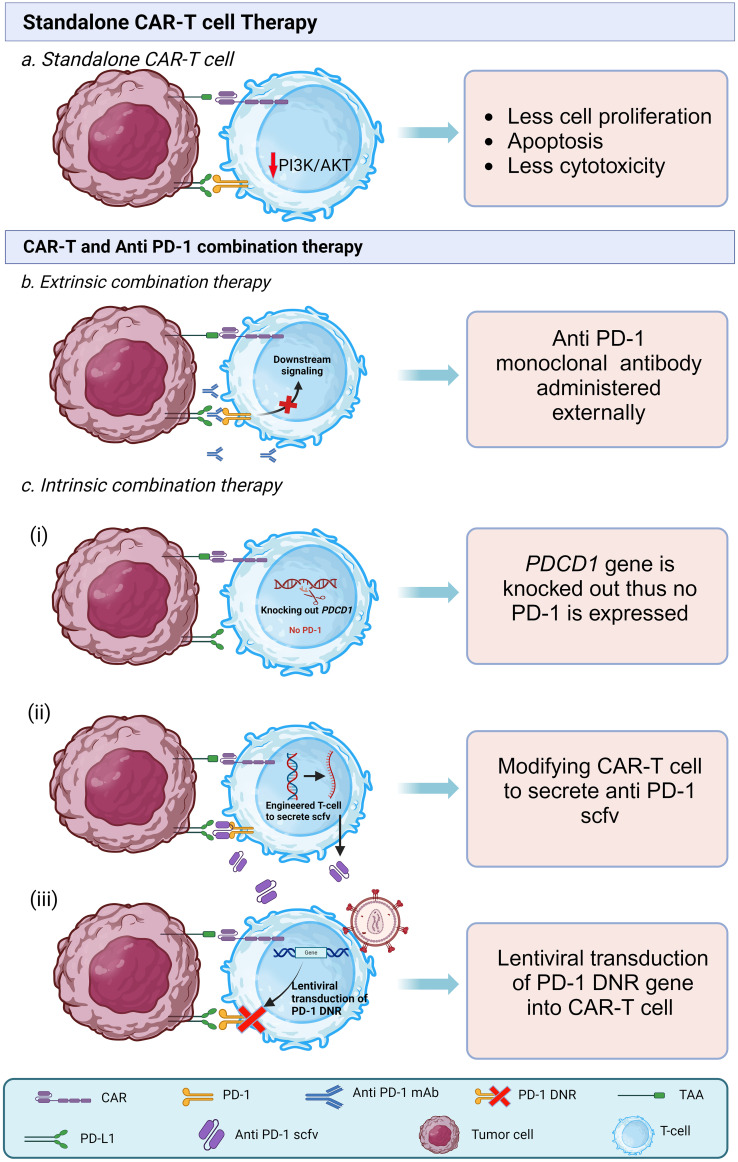
Types of CAR-T cell therapies. **(A)** Interaction of PD-1 and PDL-1 in standalone CAR-T cell in tumor microenvironment leads to low proliferation, low survival, and less cytotoxicity **(B)** In extrinsic combination therapy along with CAR-T cell anti PD-1 mAb are administered that bind to PD-1 receptor on CAR-T cell thus prevents the binding of PDL-1 with PD-1. **(C)** In intrinsic combination therapy CAR-T cells are genetically modified to prevent the activation of PD-1 downstream action. It is of three types. (i) *PDCD1* gene locus is knocked out from CAR-T cell so that it does not express PD-1 receptor. (ii) CAR-T cells are modified to secrete anti PD-1 single chain variable fragments (scFvs). (iii) PD-1 dominant negative receptor (DNR) transduced virally into the CAR-T cells, DNR lacks intracellular signaling domain.

Nonetheless, the enhanced endurance of CAR-T cells did not exhibit substantial improvement, even when combined with the natural T-cell receptor and lymphodepletion. They suggested that TCR may play a role in CAR-T cell activity (126). In an *in vitro* study by Guo, X. et al. (2018), they demonstrated that PD-1 disrupted GPC3-CAR-T cells showed stronger anti-tumor immune response compared to wild-type GPC3-CAR-T cells against PD-L1 Positive HCC cells; additionally, the disruption of PD-1 protected the CAR-T cells from exhaustion compared to wild type CAR-T cells while co-culturing (129). Another possible method of intrinsic combination is generating anti PD-1 CAR-T cells by inserting sequences that encode and secrete full-length antibodies or scFv against the PD-1 receptor (130). In a study by Fang et al. (2021) they modified autologous T-cells that contained sequences encoding scFv specific for MSLN and full-length antibody for PD-1, administered it along with small daily doses of Aptinib against advanced ovarian serous adenocarcinoma at stage IIIc and observed shrinkage in metastatic nodules, patient had progression-free survival (PFS) for five months and survived for 17 months. They found the combination therapy to be a feasible and promising treatment for advanced refractory ovarian cancer (130). Recently a novel method of cell intrinsic combination therapy is being studied that is expression of dominant negative receptor (DNR). The dominant negative receptor (DNR) has an extracellular domain of PD-1 but lacks the intracellular signaling domain of the PD-1 receptor; it acts like a decoy receptor. PD-1 DNR are prepared by inserting a sequence by viral transduction that lacks the signaling domain. CAR-T cells with PD-1 DNR show statistically significant higher proliferation rates and enhanced cytotoxicity (115). **Figure 4A** shows standalone CAR-T cell therapy **Figure 4B** shows the mechanism of cell extrinsic combination therapy and **Figure 4C** shows various types of cell intrinsic combination therapy of CAR-T cell and anti PD-1 agents.

Both types of combination therapy demonstrate encouraging efficacy, making it challenging to determine a definitive superior option. In the case of cell extrinsic combination therapy, as the anti PD-1 drugs are FDA approved, this can simplify logistics. Also, the efficacy of cell extrinsic combination therapy is encouraging. Despite this, there is a need for repeated administration of anti PD-1 drugs, and the doses and frequencies of doses still need to be generalized. In the cell intrinsic model, though, there is no need for separate dosing. However, a study found that the complete absence of PD-1 may produce terminally differentiated T-cell and can cause exhaustion of CD-8+ T-cell (131). CRISPR-Cas9 can also cause off-target mutations in Modified T-cell (132). In our opinion, using both intrinsic and extrinsic combination therapy simultaneously can show promising results, but further research and individual patient assessment are necessary to make informed treatment decisions.

### 5.2 Efficacy of anti PD-1 and CAR-T combination therapy against different types of solid tumor

Surface markers play a vital role in targeted cancer immunotherapy, and they are even more crucial in the case of solid tumors. These markers are proteins or antigens that are differentially expressed on the surface of tumor cells; thus, they have diagnostic/prognostic and therapeutic value. There are various surface markers identified to date, and they are considered a target for CAR-T cell therapy, such as mesothelin (MSLN), GD-2, CD-19, CD-30, CD-22, CD-133 (133, 134). Henceforth, we are going to discuss individual surface markers targeting combination therapy. All the studies of combination therapy discussed in this review have been summarized in **Table 2** along with clinical outcomes.

**Table 2:** Anti PD-1 and CAR-T combination therapy against different types of solid tumor.

Serial Number	Tumor type	Tumor-associated antigen	Anti PD-1 mechanism	Response	References
1	Mesothelin positive (breast cancer, lung cancer, and malignant pleural mesothelioma)	Mesothelin	Extrinsic Pembrolizumab administration	PR: 12.5% SD: 50% PD: 31.25% OS: 23.9 months	(125)
2	Mesothelin positive (pancreatic cancer, biliary tract cancer, gastric cancer, tubal cancer, esophageal cancer, ovarian cancer, cervical cancer, and triple-negative breast cancer)	Mesothelin	Intrinsic PD-1 gene disruption	SD: 46.6% OS: 3 months (4.9 months for SD patients)	(126)
3	Mesothelin positive (ovarian serous adenocarcinoma)	Mesothelin	Intrinsic PD-1 antibodies secreted by the CAR-T cells	PR: 100% PFS: 5 months	(130)
4	GD2 positive (neuroblastoma)	GD2	Extrinsic pembrolizumab administration	CR: 66.6% PR: 33.3%	(135)
5	CD19 positive (Diffuse large B-cell lymphoma, mantle cell lymphoma, transformed follicular lymphoma)	CD19	Intrinsic Chimeric switch receptor (CSR) with extracellular domain of PD-1 and signaling domain of CD28	CR: 41.2% PR: 17.6% PFS: 18 months	(136)
6	CD19/20 positive (Diffuse large B-cell lymphoma)	CD19 and CD20	Extrinsic sintilimab or camrelizumab	CR: 40% PR: 20%	(126)
7	CD-19 positive (Diffuse large B-cell lymphoma)	CD19	Extrinsic Tislelizumab	CR: 100% PFS: 18 months	(137)
8	CD19 positive (Diffuse large B-cell lymphoma)	CD19	Extrinsic sintilimab	CR: 42.3% PR: 23%	(138)
9	CD22 positive (PCNSL)	CD19 and CD22	Extrinsic camrelizumab	CR: 100%	(139)
10	CD30 positive (Hodgkin lymphoma, gray zone lymphoma, and angioimmunoblastic T-cell lymphoma)	CD30	Extrinsic anti PD-1 antibody	CR: 83.3%	(140)

CR, Complete response, PR, Partial response, SD, Stable disease, OS, Overall survival, PFS, Progression free survival.

#### 5.2.1 Mesothelin specific CAR-T cell based combination therapy

Mesothelin, a glycoprotein with a molecular weight of 40 kDa, is encoded by the MSLN gene (141). Its expression in normal cells is considerably lower compared to specific cancer cells such as pancreatic cancer, mesothelioma, ovarian and extra bile duct tumors, lung adenocarcinomas, and stomach cancers (27) This differential mesothelin expression in healthy tissues makes it an attractive target for cancer immunotherapy specific to these tumors (142). Moreover, trace amounts of mesothelin can be detected in the bloodstream of patients with mesothelin-positive cancers, making it a valuable tool for diagnosis and monitoring (143). In mesothelin-positive patients, soluble mesothelin-related peptide (SMRP), a 42–44 kDa protein, is also detected (144).

Adusumilli et al. (2021) conducted a phase I trial combining Pembrolizumab, an anti PD-1 agent, with regional mesothelin-targeted CAR-T cell therapy against patients with malignant pleural disease, including metastatic breast, lung, and malignant pleural mesothelioma (MPM) (145). In this study involving twenty-seven patients from the US, out of twenty-seven patients, twenty four had received cyclophosphamide earlier. They administered CAR-T cells intrapleurally at a dose of 0.3M - 60M CAR-T cells/kg body weight to all the patients. A significant median overall survival of 23.9 months was observed, with an average one-year survival rate of 83%. This combination therapy showed better results than the FDA-approved combination of nivolumab and ipilimumab. The combination immunotherapy also enhanced the persistence and function of CAR-T cells in peripheral blood and tumor tissue and induced new IgG responses (>3.5 fold than baseline) against tumor antigens. Sixteen patients of combination immunotherapy had measurable disease by mRECIST. The radiologic evaluation of these patients revealed partial response (PR) in two out of sixteen, stable disease (SD) in eight out of sixteen, and partial disease (PD) in five out of sixteen. The SD or better was sustained for more than six months in 8 patients; they were functionally well and did not require the subsequent treatment for a prolonged duration. They found this combination therapy safe, feasible, and effective. The study also noted reductions in serum SMRP levels post-treatment, enhanced CAR-T cell persistence and function, and induced IgG responses against tumor antigens (125).

In another phase I study led by Wang et al. (2021), PD-1 and TCR gene-disrupted CAR-T cells were assessed for their effectiveness against various solid tumors. The study included fifteen patients who underwent mesothelin-directed CAR-T cell therapy. Specifically, a mesothelin-directed 28ζ CAR-T cell was chosen, and its PDCD-1 gene and TRAC gene were knocked out using CRISPR Cas9 genome editing tools. Among the fifteen patients, six had pancreatic cancer, three had biliary tract cancer, and one each had gastric cancer, tubal cancer, esophageal cancer, ovarian cancer, cervical cancer, and triple-negative breast cancer. The treatment was found to be safe, with no observed neurotoxicity or autoimmune reactions. However, out of the fifteen patients, only seven exhibited stable disease (SD) three to four weeks after the infusion. Unfortunately, this response was sustained in only two patients until the subsequent follow-up after eight to twelve weeks. The median overall survival for all fifteen patients was 3.0 months, while for the seven patients showing stable disease at weeks 3–4 post-infusion, it was 4.9 months. The study attributed the relatively poor outcome to the disruption of the TCR, which had a negative impact on CAR-T cell proliferation (126).

Furthermore, Fang J et al. presented a case study involving a 54-year-old female diagnosed with advanced ovarian serous adenocarcinoma. The immunohistochemical staining revealed increased mesothelin expression in tumor cells (MSLN +++84%). To address advanced ovarian serous adenocarcinoma, T-cells were co-cultured with mesothelin antigen, anti-CD28, and recombinant human IL-2 to prepare αPD-1-meso CAR-T cells. These cells were then administered to the patient via intravenous drip on day 0 and day 26. Additionally, the patient received daily aptinib at a dose of 250 mg, known for its antiangiogenic properties and ability to enhance the efficacy of immune checkpoint inhibitors (ICI).

Flow cytometry analysis showed no change in total leukocyte count but a shift in lymphocyte count. Specifically, CD8+ T-cell count increased to 522 and 612 cells/µl at months one and three, respectively, compared to 352 cells/µl before CAR-T therapy. Conversely, CD4+ T-cell count decreased to 512 and 522 cells/µl at months one and three, respectively, compared to 800 cells/µl before CAR-T therapy. These findings suggested that αPD-1-meso CAR-T cells contributed to the activation of CD8+ T-cells. Notably, one tumor nodule decreased in size from 51.9 mm to 39.1 mm, while another nodule became undetectable. Based on Response Evaluation Criteria in Solid Tumors V.1.1, the patient exhibited partial response (PR) and showed shrinkage of nodules and progression-free survival for five months, demonstrating the effectiveness and feasibility of this immunotherapy against mesothelin-positive refractory breast cancer (130).

#### 5.2.2 GD-2 specific CAR-T cell based combination therapy

GD-2, also referred to as disialoganglioside GD2, is a type of glycosphingolipid found abundantly on the surfaces of tumor cells but with limited expression in normal cells. This characteristic makes it an appealing target for cancer immunotherapy (28), particularly in the treatment of neuroblastoma (NB), a prevalent extracranial solid tumor in children (146). Monoclonal antibodies designed to target GD2 have become part of standard care for high-risk neuroblastoma patients, despite the significant toxicities they may cause (135). As an alternative approach, researchers are exploring GD2-targeted CAR-T cell therapy.

Heczey et al. (2017) conducted a Phase I trial involving eleven patients with relapsed or refractory neuroblastoma, with a median age of 6.5 years. All patients received third-generation GD2-CAR-T cells incorporating CD28 and OX40 costimulatory endodomains. Ten out of eleven patients had stage 4 (metastatic) NB, while one had stage 3 NB located in the pelvis region; all patients had active disease at the time of CAR-T infusions. The patients were divided into three cohorts for comparison.

In Cohort 1, four patients received only GD-2 CAR3-T. Cohort 2 comprised four patients who received GD-2 CAR3-T along with cyclophosphamide and fludarabine (Cy/Flu) preconditioning to enhance CAR-T expansion and persistence. In Cohort 3, three patients received both Cy/Flu and GD-2 CAR3-T, along with pembrolizumab, an anti PD-1 agent. The doses of GD2-CAR3-T Cells were calculated based on body surface area according to the INSS International Neuroblastoma Staging System. Cyclophosphamide was administered at 500 mg/m2/dose on days -4, −3, and −2, and fludarabine at 30 mg/m2/dose on days −4 and −3 intravenously, while pembrolizumab was given at 2 mg/kg/dose intravenously on the -1st and 21st day of CAR-T administration. Among the eleven patients, only one patient in Cohort 1 experienced cytokine release syndrome, which did not exceed grade 2.

While there was no significant difference observed between Cy/Flu, GD-2 CAR3-T patients and anti PD-1, Cy/Flu, GD-2 CAR3-T patients in terms of T-cell persistence and expansion, the best response was noted in Cohort 3. In this cohort, which involved anti PD-1 administration, two out of three patients exhibited complete response, while one showed partial disease response. In Cohort 1, out of four patients, two had stable disease, and two showed progressive disease. In Cohort 2, out of four patients, three had partial disease, and only one showed stable disease. These findings suggest that combining checkpoint inhibitors with CAR-T cells might result in improved patient responses, although further studies with larger patient cohorts are warranted to validate these observations (135).

#### 5.2.3 CD-19 directed CAR-T cell based combination immunotherapy

CD-19, a protein weighing 95 kDa, is part of the immunoglobulin superfamily and is primarily located on the surface of B lymphocytes. It acts as a co-receptor for the B cell antigen receptor (BCR) and plays a crucial role in B-cell signaling by enhancing cell proliferation, mitogen-activated protein kinase activity, and calcium release synergistically in B-cells when co-ligated with the BCR (147).B-cell lymphoma, cancer affecting lymphocytes, can present as a solid tumor in lymph nodes or other tissues, involving the bone marrow and circulating in the blood (29).

In a phase Ib study by Liu et al. (2021), six PD-L1 positive B-cell R/R DLBCL lymphoma patients were treated with CD19-PD-1/CD28-CAR-T cells derived from autologous blood via leukapheresis (148). Among these patients, three achieved complete response (CR), one had stable disease, while two succumbed to progressive disease by day 60. Of the three patients with CR, two maintained remissions up to the cutoff date, and one showed remission after 12 months. Although anti-CD19-CAR-T cell therapy showed promising results, the main contributor to CAR-T remission against DLBCL was found to be the expression of PD-L1 on tumor cells. Consequently, in subsequent research, authors incorporated a chimeric switch-receptor (CSR) where the truncated extracellular domain of PD-1 binds to cytoplasmic signaling domains of CD28, facilitating T-cell activation. When PD-L1 binds to the PD-1 of CSR, it activates downstream CD28 signaling (149).

This study involved seventeen Chinese patients with a median age of 55, including thirteen with DLBCL, two with mantle cell lymphoma (MCL), and two with transformed follicular lymphoma (tFL). All patients received chemotherapy as conditioning before administration of CD19-PD-1/CD28-CAR-T cells at doses ranging from 0.5 to 4.0 × 10^6 CAR-T cells/kg. During the initial four weeks, all patients experienced one or more adverse events (AE) of grade 3 or 4, with leukopenia (88.23%), thrombocytopenia (35.29%), anemia (23.53%), and pyrexia (23.53%) being the most frequent. Thirteen patients (88.24%) had moderate CRS symptoms, managed with supportive therapy. Seven patients (41.17%) experienced hypotension, and one patient (5.88%) had hypoxia, all treated with appropriate measures.

Despite patient 17’s unfortunate death due to lymphoma progression on day 30, without severe CRS or CAR-T related neurotoxicity, the study found no significant correlation between the degree of adverse events and PD-L1 tumor expression. Ten of seventeen patients (58.8%) achieved an objective response (CR or PR) at three months, with seven (41.2%) reaching CR, including one patient each with MCL and tFL. Among those with CR or PR at three months, 80% had progression-free survival (PFS) at eighteen months. While these findings are promising, further research is necessary to mitigate adverse events (136).

Wang et al. (2021) investigated a study involving five patients with relapsed/refractory (R/R) DLBCL, with a median age of 41 years, who had previously undergone CD19/20-CAR-T therapy but experienced disease progression afterward (126). In an effort to enhance efficacy, a combination therapy of CD19/20-CAR-T and PD-1 blockade therapy was administered to these patients. Participants received 200 mg of PD-1-blocking antibodies, such as sintilimab or camrelizumab, every two weeks until disease progression or intolerable toxicity occurred. The anti-tumor response was monitored using PET/CT imaging. Notably, no CRS or toxicities ≥ grade 3 were observed. Among the five patients, three achieved objective responses following PD-1 therapy, with two achieving complete response (CR) and one achieving partial response (PR). Unfortunately, the remaining two patients experienced disease progression and succumbed to their conditions between 15.6 and 16.2 months after treatment. Patient 4 maintained CR up to the cutoff date, which was 21.3 months post-treatment, with a progression-free survival (PFS) of 21.8 months. Additionally, one patient exhibited complete elimination of the baseline tumor, although a new lesion emerged ten months later. The study suggests that PD-1 blockade therapy may be effective for patients with relapsed or refractory DLBCL who did not respond adequately to CAR-T cell therapy and who exhibit high levels of PD-1 in T-cells infiltrated by tumors (126).

A case report by Zhang et al. (2022) examined a 38-year-old female patient with relapsed DLBCL of the CNS and TP53 mutation. Initially diagnosed with stage IIIA GCB-DLBCL, the patient underwent chemotherapy alongside antiviral therapy due to a history of hepatitis B and detectable HBV DNA levels. Four months into chemotherapy, the patient experienced her first relapse, diagnosed as stage IVA GCB-DLBCL, prompting immediate chemotherapy. However, after two months, the disease relapsed again. Given the circumstances, the patient was enrolled for CD19 CAR-T therapy, receiving lymphodepleting therapy in addition to CAR-T therapy.

Following infusion, monitoring of CD-19 CAR-T cell DNA copy levels revealed counts of 4.6×10 copies/µg on day 0 and 5.65×10 copies/µg on day 7. On day 8, due to the patient’s symptoms, a Bruton Tyrosine Kinase (BTK) inhibitor, Zanubrutinib (160 mg), was administered alongside CAR-T therapy. Additionally, on day 15, the PD-1 inhibitor Tislelizumab (200 mg) was introduced to improve the patient’s condition. However, the patient experienced menorrhagia and severe anemia, necessitating an RBC transfusion.

One month later, further complications arose as the patient was diagnosed with stage II endometrial carcinoma. Subsequent radiation treatment for endometrial cancer was delayed due to the Covid-19 pandemic. Nonetheless, follow-up examinations indicated no recurrence of symptoms, and complete remission (CR) was achieved approximately 18 months following CD19 CAR-T cell treatment (137).

Mu et al. (2022) conducted a study involving forty-four patients with relapsed/refractory (R/R) DLBCL to assess the safety and efficacy of combining PD-1 inhibitors with anti-CD19 CAR-T cell therapy, followed by PD-1 inhibitor maintenance therapy in DLBCL patients with high tumor burden (138). These patients were divided into two groups: a combination group of twenty-six patients and a control group of eighteen patients. Lymphodepleting chemotherapy was administered to all patients, consisting of fludarabine (30 mg/m2/day) and cyclophosphamide (400 mg/m2/day) between days 4 and 2. On day 1, patients in the combination group received PD-1 inhibitors (Sintilimab, 200 mg). The anti-CD19 CAR-T cell infusion dose on day 0 for all forty-four patients with R/R DLBCL was 2 × 10^6 cells/kg. The efficacy of these therapies was evaluated at one- and two-months post-infusion.

In the combination group, eleven out of twenty-six patients achieved complete response (CR) (42.31%), while six achieved partial response (PR) (23.08%), resulting in an objective response rate (ORR) of 65.39%. In comparison, in the control group, seven patients (38.89%) achieved CR, four (22.22%) had PR, four (22.22%) had stable disease (SD), and three (16.67%) had progressive disease (PD). The ORR in the control group was 61.11%. The study suggests that combination therapy yields promising results; however, it may entail some side effects, particularly in the case of lymphomas (138).

#### 5.2.4 CD-22 specific CAR-T cell based combination immunotherapy

CD-22 is a transmembrane protein weighing 140 kDa, primarily found in mature B-cell lineages, where it acts as a coreceptor of the B cell receptor (BCR) (30). The CD-22 gene, located on q13.1 of chromosome 19, spans around 22kb of DNA and consists of 13 exons (150).

Zou et al. (2023) reported a case involving a 49-year-old male patient with primary central nervous system lymphoma (PCNSL). Initially treated with a combination of 375 mg/m2 rituximab and 3.5 g/m2 methotrexate (MTX), the patient exhibited no improvement. Subsequent MRI scans revealed new lesions, prompting a shift to cytarabine (2 g/m2 q12h on days 1 and 2), with no significant response. Treatment was then transitioned to targeted therapies, including ibrutinib and lenalidomide, followed by three doses of the PD-1 inhibitor camrelizumab, yet still without improvement. Finally, the patient underwent CAR-T therapy.

Immunohistochemical staining confirmed the patient’s positivity for CD19/CD22. Peripheral blood mononuclear cells were collected and utilized to prepare second-generation CAR-T cells. Before CAR-T administration, the patient underwent chemotherapy with the DCF regimen (consisting of intravenous administration of 100 mg/m2 Decitabine for three consecutive days, 300 mg/m2 cyclophosphamide on days 1 to 3, and 30 mg/m2 fludarabine on days 1 to 3) to reduce lymphocytes. Subsequently, CD19 and CD22-specific CAR-T cells were infused at a total dose of 1 million CAR-T cells per kilogram of the patient’s body weight (1×10^7/kg), with three dose escalations of 10%, 30%, and 60% post-infusion. The patient experienced grade 2 cytokine release syndrome, which was managed without posing any imminent threat. One month post-CAR-T infusion, consistent levels of CAR-T cells ranging from 987 to 2,340,000 copies per microgram of DNA were observed in the patient’s peripheral blood. An MRI scan at this juncture revealed complete remission (CR). To prevent relapse, the patient received 200 mg of camrelizumab (a PD-1 inhibitor) every three weeks, along with daily administration of 560 mg of ibrutinib. Thankfully, the patient remains alive, demonstrating the efficacy and potential of CAR-T therapy in such cases (139).

#### 5.2.5 CD-133 specific CAR-T based combination immunotherapy

CD133, a transmembrane glycoprotein weighing 97 kDa, is encoded by the prominin 1 (PROM1) gene located on chromosome 4 in humans (31). It has been identified as a marker for cancer stem cell (CSC) populations across various solid tumor types, including brain cancer (151), prostate cancer (152), colon cancer (153), lung cancer (154), hepatocellular carcinoma (HCC) (155), and ovarian cancer (156, 157).

Hepatocellular carcinoma (HCC) stands as the most prevalent form of liver cancer and ranks as the third leading cause of cancer-related mortality globally (2). Current therapies for advanced HCC, such as sorafenib, regorafenib, and lenvatinib, offer only modest improvements in overall survival (158). Notably, recently licensed PD-1 inhibitors such as pembrolizumab and nivolumab have exhibited inadequate response rates, underscoring the pressing need for more effective treatment options. CD133, associated with HCC stem cell-like cells, is linked to advanced tumors and poor prognosis, rendering it an appealing target for therapy. Chimeric antigen receptor-specific T (CAR-T) cells specifically targeting CD133-positive CSCs have emerged as a promising treatment modality for advanced HCC, offering potential advancements in the therapeutic landscape for this challenging disease.

Yang et al. (2023) conducted a preliminary study to assess the feasibility of utilizing a CD133-specific CAR-T cell system to deliver locally PD-1 blocking scFv as a monotherapy for advanced hepatocellular carcinoma (HCC). The study included 67 HCC patient samples, categorized into stages, and twenty-six cases of stage IV lung metastasis. Higher CD133 expression was detected in nineteen out of sixty-seven primary HCC tissues, correlating significantly with portal vein invasion and AFP levels. However, there was no statistically significant relationship between CD133 expression and overall survival (OS) or progression-free survival (PFS) in the entire cohort. Notably, in late-stage (II and III) male patients, CD133 expression was significantly associated with poorer PFS and OS, consistent with findings from online TCGA data.

To evaluate the effectiveness of CD133 as a target, CAR-T cells were engineered with a custom plasmid containing an anti-CD133 scFv gene segment derived from HW350341.1, a c-Myc tag gene segment, a CD8TM gene segment, and a secretory PD-1 blocking scFv. These engineered CAR-T cells were then transduced using sleeping beauty-mediated transposition.

In vivo, experiments were conducted using NCG mice injected with Hep-3B-luc tumor cells to establish subcutaneous and *in situ* xenograft tumor models. Mice were treated with Mock T-cells, CD133 CAR-T cells alone, or PD-1 inhibitor-secreting CD133 CAR-T cells once tumors reached a size of 50–100 mm3. Intraperitoneal injections were administered, and tumor progression was monitored using bioluminescence imaging (BLI).

The study demonstrated that PD-1 inhibitor-secreting CD133 CAR-T cells significantly extended the survival of mice compared to CD133 CAR-T cells and Mock T-cells. Tumor BLI showed that PD-1 inhibitor-secreting CD133 CAR-T cells exhibited more pronounced antitumor activity compared to Mock T-cells and CD133 CAR-T cells alone. Additionally, a higher proportion of human T-cells was observed within the CD133 CAR-T tumor microenvironment and the PD-1 inhibitor-secreting CD133 CAR-T group compared to the Mock T group. Overall, these findings suggest that combining PD-1 blockade with CD133-specific CAR-T cell therapy holds promise as a feasible treatment approach for advanced HCC (159).

#### 5.2.6 CD-30 specific CAR-T based combination therapy

CD30 is a type 1 transmembrane glycoprotein belonging to the tumor necrosis factor receptor superfamily 8 (TNFRSF), with a molecular weight ranging from 105 to 120 kDa. It is encoded by the CD30 gene located on chromosome 1p36.2-26.3. Initially identified on Hedgehog and Reed-Sternberg cells of Hodgkin lymphoma, it was termed Ki-1 (160–162). The high expression of CD30 is commonly observed in Hodgkin lymphoma (HL) and systemic anaplastic large cell lymphoma (ALCL) (163, 164). In addition to HL and ALCL, CD30 expression is also observed in a subset of diffuse large B cell lymphoma (DLBCL), albeit infrequently, as well as in follicular lymphomas (FL), primary cutaneous anaplastic lymphomas, and the lymphomatoid papulosis form of cutaneous T-cell lymphoma. Furthermore, certain non-hematological malignancies, such as embryonal carcinoma and specific seminomas, also express CD30. These diverse expressions of CD30 highlight its significance as a diagnostic and therapeutic target across various lymphoproliferative disorders and certain non-hematological malignancies (32, 165–167).

Sang et al. (2022) conducted phase II trials to assess the efficacy of combination therapy involving anti-CD30 directed CAR-T cells and anti PD-1 antibodies in cases of relapsed and refractory CD30+ lymphomas, including Hodgkin lymphoma (HL), gray zone lymphoma (GZL), and angioimmunoblastic T-cell lymphoma (AITL) of various grades. Three cohorts comprising a total of twelve patients were included in the study. In Cohort 1, patients received 10^6/kg CAR-T cells, while in Cohort 2, patients received 10^7/kg CAR-T cells. Cohort 3 patients received additional anti PD-1 antibodies starting 14 days after infusion of 10^7/kg CAR-T cells, administered every three weeks after that. The results showed that three out of four patients in Cohort 1 achieved partial response (PR), while two out of three patients in Cohort 2 achieved complete response (CR), with one patient achieving PR. In contrast, all patients in Cohort 3 showed a 100% objective response rate (ORR), with 80% achieving CR and exhibiting low toxicity levels. Furthermore, of the eleven patients who responded to CAR-T therapy, seven remained responsive until October 31, 2021, and four out of the six patients who achieved CR maintained their CR status. Notably, among HL patients receiving 10^7/kg CAR-T cells plus PD-1-blocking antibody, five out of six (83.3%) showed CR, while patients receiving 10^6/kg CAR-T cells (Cohort 1) did not achieve CR. In conclusion, the study demonstrated that CAR-T therapy and PD-1 blockade yielded better outcomes than CAR-T therapy alone, with a higher rate of complete responses observed, particularly in HL patients (140).

## 6 Conclusion and outlooks

Currently, the FDA has approved CAR-T cell therapy as a standalone treatment for several hematological malignancies such as acute lymphoblastic leukemia (ALL), large B cell lymphoma (LBCL), follicular lymphoma (FL), mantle cell lymphoma (MCL), marginal zone lymphoma (MZL), and multiple myeloma (MM). Particularly in hematological malignancies, CAR-T cell therapy stands out as an effective therapeutic option, with a remarkable overall response rate (ORR) of up to 97% and 67% complete remission (CR) for the product Cilta-cel in multiple myeloma (MM) (168).

However, the application of CAR-T cell therapy for solid tumors poses significant challenges, including tumor infiltration and trafficking difficulties, cytokine release syndrome, on-target off-tumor toxicity, and T-cell exhaustion within the complex tumor microenvironment (38, 169–172).Till date, various types of combination immunotherapies have been studied, such as CAR-T cell with anti PD-L1 (173), double immune checkpoint inhibitor therapy such as anti PD-1/PD-L1 plus anti-CTLA-4 (174), and CAR-T cells with anti PD-1 (175). In a study by Cheng et al. (2023) combination of 4-1BB CAR-T and autocrine anti PD-L1 scFv improved the anti-tumor response of CAR-T cells along with improved persistence. However, the study was conducted in a mouse xenograft model and needs to be conducted in clinical patients for a better assessment of actual outcomes (176). The double checkpoint blockade strategy, particularly the combination of nivolumab (anti-PD-1 mAb) and ipilimumab (anti-CTLA-4), received FDA approval in October 2020 as a first-line treatment option (145), a meta-analysis by Zhao et al. (2022) revealed that five out of the ten patients receiving nivolumab in addition to ipilimumab experienced at least one grade ≥ 3 adverse event, and approximately nine of the ten patients experienced at least one adverse event. The most frequent mild adverse event among them was fatigue (30.92%), while the most frequent grade ≥ 3 adverse event was elevated ALT (8.12%) (177). However, in contrast to the above-mentioned combination therapies, CAR-T cell and anti PD-1 therapy have been studied in patients, and in the case of various solid tumors such as mesothelioma, as discussed earlier, even yield better results than double immune checkpoint inhibitor therapy (125). Combining CAR-T cell therapy with PD-1 blockade has been explored to address these challenges and enhance the effectiveness of cancer treatment. This combination synergistically addresses the limitations of each approach individually, such as mitigating side effects and counteracting the impact of the tumor environment on treatment outcomes (71). Encouraging findings suggest that this combination improves progression-free survival and partial and complete responses, potentially enhancing overall survival rates.

This review provides a comprehensive overview of the improved efficacy observed with combined CAR-T therapy and PD-1 blockade in lymphomas and solid tumors. Detailed examinations of various tumors indicate positive outcomes, with the combination therapy demonstrating promising results in targeted mesothelin-positive solid tumors, GD-2 expressing neuroblastoma, CD-19-positive tumors, CD-22-positive tumors, CD-30-directed therapy, and CD133-directed CAR-T cell-based combination therapy. The targeted mesothelin-positive solid tumors showed that the externally given (extrinsic) combination therapy of Pembrolizumab, and CAR-T cell delivered an encouraging result that is an overall survival of 23.9 months. In the case of GD-2 expressing neuroblastoma the combination therapy of CAR-T with pembrolizumab yielded the best result compared to standalone CAR-T cells. In the case of CD-19-positive tumors, a combination of CAR-T and anti PD-1 showed that almost half of the patients had median PFS for about 18 months and 21.8 months in two studies. In the case of CD-22 positive tumor, the patient showed complete remission after the administration of CAR-T and maintenance of anti PD-1 and the patient had progression-free survival (PFS) of 35 months till the cutoff date. In CD-30-directed combination therapy, more than 80% of patients showed CR in the case of CAR-T and anti PD-1 combination therapy. CD133-directed CAR-T cell-based combination therapy also showed better results than stand-alone CAR-T. Overall in several cancer, CR rates, median progression-free survival (PFS), and overall survival outcomes have improved.

Despite these promising results, targeting specific antigens with CAR-T cells faces limitations due to diverse antigen expression in solid tumors. The combination of CAR-T and anti PD-1 therapy might not be sufficient to accomplish the T cell infiltration and effector function required to successfully combat solid tumors (71). Solid tumors may develop immune evasion strategies or alter antigen expression as resistance mechanisms, potentially reducing the duration of action of combination therapy. Additionally, assessing the long-term efficacy, durability, and potential late-onset adverse events of combination therapy remains challenging due to a lack of comprehensive long-term safety and effectiveness data over extended periods. The high combined costs of CAR-T cell therapy with PD-1 blocking may pose affordability issues for many patients. The current available FDA and European Medicines agency-approved CAR-T cell therapies are autologous and derived from the patient’s blood through leukapheresis. While these therapies have benefits such as reduced risk of rejection and longer persistence in the patient’s system, they also come with drawbacks such as high costs, extensive processing time (often up to three weeks), and variable outcomes due to individual differences in cellular quality (178). To address these limitations, researchers are exploring the development of allogeneic CAR-T cell therapies, also called “off-the-shelf” treatment, which can be manufactured from the T-cells of healthy donors and offer benefits such as cost-effectiveness and immediate availability. However, allogeneic therapies also carry the risk of Graft-versus-Host Disease (GVHD) due to HLA disparity (179). In a recent phase I clinical trial (NCT04538599), Hu et al. (2022) successfully administered allogeneic CAR-T cells to treat CD7-positive hematologic malignancies. The modified cells were engineered to eliminate CD7, T cell receptor (TCR), and human leukocyte antigen (HLA) class II expression and to express a natural killer (NK) cell inhibitor and the common cytokine receptor gamma chain (γc). The trial showed promising results, with 81.8% of participants exhibiting objective responses and a complete response rate of 63.6% (180). Ongoing research is focused on developing a universal CAR-T cell therapy using human-induced pluripotent stem cells (iPSCs), which could offer an off-the-shelf option without the associated adverse events and processing time (178, 181).

To establish a standardized protocol for all tumors, it is crucial to specify the optimal combination therapy protocol and dosage for CAR-T cell and PD-1 blockade. Addressing side effects necessitates the development of targeted delivery systems and the integration of additional agents. Research efforts should explore novel approaches, such as creating iPSC-based universal CAR-T cells, to overcome difficulties associated with using patients’ autologous CAR-T cells.

We believe that the synergistic approaches discussed in this review will contribute valuable insights, paving the way for developing a promising immunotherapy in the future.

## Data availability statement

The original contributions presented in the study are included in the article/supplementary material, further inquiries can be directed to the corresponding author/s.

## Author contributions

BPS: Data curation, Investigation, Methodology, Visualization, Writing – original draft. PS: Data curation, Investigation, Methodology, Visualization, Writing – original draft, Conceptualization. RoY: Data curation, Investigation, Methodology, Visualization, Writing – original draft. DC: Data curation, Investigation, Methodology, Visualization, Writing – original draft. DS: Data curation, Investigation, Methodology, Writing – original draft. CPD: Data curation, Investigation, Methodology, Writing – original draft. PD: Data curation, Investigation, Methodology, Writing – original draft. VU: Data curation, Investigation, Supervision, Writing – review & editing. RiY: Writing – original draft. MJ: Conceptualization, Funding acquisition, Project administration, Resources, Supervision, Writing – review & editing. AJ: Conceptualization, Project administration, Resources, Supervision, Writing – review & editing.
